# Supplementary material for: Bacterial Isolates Derived from Nest Soil Affect the Attraction and Digging Behavior of Workers of the Red Imported Fire Ant, Solenopsis invicta Buren
Source: Insects. 2022 May 7;13(5):444. doi: 10.3390/insects13050444 (PMC9145412; doi:10.3390/insects13050444)
Supplement: Supplementary file 1 [file insects-13-00444-s001.zip › insects-1655554-supplementary.pdf]

Supplementary data

Methods S1

To determine the minimum number of RIFA needed to detect sand excavation at 18 h, additional 2-choice bioassays were conducted, each providing control sand as both choices. Worker ants in varying numbers (1, 2, 4, 6, 8, 10, 15, and 20) were introduced into the assay arena and allowed to excavate sand for 18 h. For 1, 2, 4, 6, 8, and 10 ants per assay, four trials were conducted with polygyne RIFA and four trials with monogyne RIFA. For the 15 and 20 ants per assay, eight trials were conducted with polygyne RIFA and eight trials with monogyne RIFA. Subsequently, the total amount of sand excavated (sum of sand from both choices) was determined and compared to the total sand displaced in the dummy assays (0 ants/assay) that were previously described. Student's *t*-tests ($\alpha = 0.05$) were conducted to compare the average total amount of sand displaced in both assays.

Results S2

An average (±SE) of 7.9 (5.3) mg of sand was displaced in dummy assays that included 0 RIFA workers. Similar amounts of displaced sand were measured in assays having 1, 2, and 4 RIFA, each of which showed no significant difference (*t*-test, $P > 0.05$) compared to the 0 RIFA group (Table S1). Assays with 6, 8, 10, 15, and 20 RIFA, however, were significantly different (*t*-test, $P < 0.05$) from the 0 RIFA dummy assays (Table S1). Assays with six RIFA had the fewest RIFA workers needed to give significantly different results from the 0 RIFA assays. Six RIFA workers per assay were, therefore, used in time-course behavioral assays.

Table S1. Total amount of sand excavated in bioassays performed with varying numbers of RIFA workers

Number of ants per assay	Average (±SE) total sand displaced (mg)	*n*	$p > t$*
0	7.9 (5.3)	32	-
1	18.9 (5.2)	8	0.8940
2	6.4 (1.8)	8	0.9850
4	10.3 (5.3)	8	0.9777
6	194.9 (77.6)	8	**0.0258**
8	263.8 (49.9)	8	**0.0025**
10	395.4 (124.4)	8	**<0.0001**
15	606.6 (72.1)	16	**<0.0001**
20	741.4 (89.1	16	**<0.0001**

*Comparisons (*t*-test, $\alpha = 0.05$) of the quantities of sand displaced in 0 RIFA per assay with >0 RIFA per assay to determine the minimum number of RIFA per assay needed to detect sand displaced by ant digging activity.
